# Cellular Lyso-Gb3 Is a Biomarker for Mucolipidosis II

**DOI:** 10.3390/ijms26136275

**Published:** 2025-06-29

**Authors:** Seigo Terawaki, Hiroki Nakanishi, Toko Shibuya, Norio Sakai, Takanobu Otomo

**Affiliations:** 1 Department of Molecular and Genetic Medicine, Kawasaki Medical School, Kurashiki 701-0192, Japan; 2 Lipidome Lab Co., Ltd., Akita 010-0825, Japan; 3 Department of Pediatrics, University of Osaka, Suita 565-0871, Japan

**Keywords:** mucolipidosis, Lyso-Gb3, biomarker, lysosomal storage disease

## Abstract

Lysosomal storage diseases are caused by defective lysosomal function, such as impaired lysosomal enzyme activities, which include more than 70 different diseases. Although biomarkers and therapies have been developed to date for some of them, many others remain challenging to diagnose and treat. In this study, an elevated level of Globotriaosylsphingosine (Lyso-Gb3), an already known biomarker for Fabry disease, was confirmed in the knock-out cells of the *GLA*, *GNPTAB*, and *PSAP* genes and models for Fabry, mucolipidosis II/III (ML II/III), and combined saposin deficiency, respectively. Lyso-Gb3 was high in ML II/III patient skin fibroblasts compared with normal cells and was decreased after total lysosomal enzyme supplementation. There have been no useful biomarkers reported in ML II/III until now. This study shows that Lyso-Gb3 is elevated in ML II/III patient cells and is decreased by treatment, indicating that Lyso-Gb3 is a potential biomarker for ML II/III.

## 1. Introduction

Lysosomal storage diseases (LSDs) are inborn errors of metabolism caused by defective lysosomal functions. LSDs include more than 70 diseases, many of which are caused by lysosomal enzyme deficiency, and the others contain impairments of lysosomal targeting of lysosomal hydrolases, lysosomal transporters, lysosomal environments for degradation, and so on [1].

Lysosomal dysfunction leads to the accumulation of undegraded substrates, resulting in a variety of clinical phenotypes. Deficiency of a lysosomal enzyme leads to the primary accumulation of the enzyme’s substrates. Furthermore, in lysosomes filled with a massive accumulation of undegraded substrates, various hydrolytic enzymatic reactions are thought to be impaired, resulting in secondary accumulation of various substrates due to a general decline in lysosomal function. Indeed, we have reported the disruption of the lysosomal acidic environment in mucolipidosis II/III (ML II/III) and lysosomal acid lipase deficiency (LAL-D) [2,3].

The disruption of intracellular vesicular trafficking is also known to cause LSDs. We reported that a genetic mutation in *VPS33A*, which is a component of protein complexes for tethering intracellular vesicles, leads to mucopolysaccharidosis-plus syndrome [4]. This novel disease manifests a similar clinical phenotype to LSDs and is speculated to be due to impaired transport of substrates to be degraded by lysosomes [5]. ML II/III is caused by multiple deficiencies of lysosomal enzymes due to the disruption of intracellular trafficking of lysosomal enzymes. In ML II/III, abnormalities in the intracellular transport of lipids and in the distribution of mannose 6-phosphate (M6P) receptors that function in lysosomal enzyme transport are reported [2].

These complex mechanisms of lysosomal dysfunction make it difficult to objectively evaluate the effectiveness of specific treatments for LSDs. Appropriate biomarkers that precisely reflect the severity and progression of diseases, or the effectiveness of therapies, are indispensable for the diagnosis and future development of treatments for LSDs such as ML II/III, in which no valuable biomarkers are available.

In this study, we focused on one of the sphingolipids, Globotriaosylsphingosine (Lyso-Gb3). Lyso-Gb3 is a known biomarker for the diagnosis and disease assessment of Fabry disease, a type of LSD [6,7,8,9]. Fabry disease is caused by a deficiency in the *GLA* gene that codes for α-galactosidase A, a lysosomal hydrolase. Sphingolipids such as globotriaosylceramide (Gb3) and galactosylceramide are the substrates of α-galactosidase A and accumulate in tissues throughout the body. In particular, Lyso-Gb3, the lyso form of Gb3, has been suggested to be related to the organ damage caused by Fabry disease, such as by promoting the proliferation of smooth muscle, thickening vascular walls, and damaging renal podocytes [10]. Therefore, considering the pathological overlap in sphingolipidoses and other LSDs, we decided to measure Lyso-Gb3 cross-sectionally in LSD model cells with uniform genetic backgrounds and in patient specimens to clarify the profile of Lyso-Gb3 accumulation.

## 2. Results and Discussion

**Figure 1:**
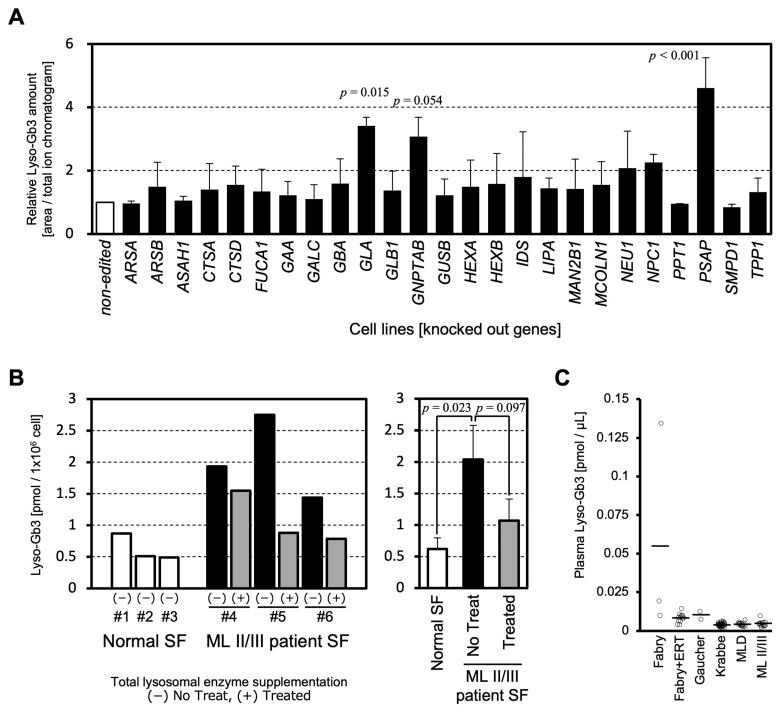
Lyso-Gb3 in LSDs. (**A**) Relative amount of Lyso-Gb3 in LSD model cells. Values “area/total ion chromatogram” were obtained by LC-MS/MS in each cell line. Labels at the bottom indicate genes knocked out in HeLa cells, except for the non-edited cell line. Data from three different genotype clones (Table 1). Values are normalized to non-edited HeLa cells, and the mean and SDs are shown. The absolute quantification values of Lyso-Gb3 from the non-edited and *GNPTAB* KO cells were measured in a separate experiment to be 0.30 and 0.70 pmol/10^6^ cells, respectively. Statistical significances between all KO cell lines were tested using one-way ANOVA, followed by Dunnett’s post hoc test for comparison with the control group. Only *p*-values less than 0.1 are shown on top of the bar graph. (**B**) Lyso-Gb3 amount in skin fibroblasts (SFs). Three normal SFs (#1–3) and three ML II/III patient SFs (#4–6) were analyzed for Lyso-Gb3. Parts of the ML II/III skin fibroblasts were treated with a total lysosomal enzyme mixture. The right panel represents the mean values and SDs for the normal and patient SFs (the no treat group and the treated group) from the left panel. Statistical analysis was performed using one-way ANOVA followed by Tukey’s post hoc test among the three groups of samples. *p*-values less than 0.1 are shown. (**C**) Lyso-Gb3 amount in patient plasma. Each data point is indicated by a small circle. The horizontal bars represent the means for each data group. Fabry patients without enzyme replacement therapy (ERT) (*n* = 3); Fabry patients undergoing ERT (*n* = 12); Gaucher patients (*n* = 2); Krabbe patients (*n* = 33); metachromatic leukodystrophy (MLD) patients (*n* = 15); and ML II/III patients (*n* = 14).

To systematically compare whether lysosomal dysfunction in various LSDs affects lipid metabolism and leads to Lyso-Gb3 accumulation, we established disease model cells by knockout (KO) of 25 lysosomal enzymes or lysosome-related genes using CRISPR/Cas9 in the HeLa cell line: *ARSA* KO (metachromatic leukodystrophy), *ARSB* KO (mucopolysaccharidosis VI), *ASAH1* KO (Farber disease), *CTSA* KO (galactosialidosis), *CTSD* KO (neuronal ceroid lipofuscinosis 10), *FUCA1* KO (fucosidosis), *GAA* KO (Pompe disease), *GALC* KO (Krabbe disease), *GBA* KO (Gaucher disease), *GLA* KO (Fabry disease), *GLB1* KO (GM1 gangliosidosis), *GNPTAB* KO (ML II/III), *GUSB* KO (mucopolysaccharidosis VII), *HEXA* KO (Tay-Sachs disease), *HEXB* KO (Sandhoff disease), *IDS* KO (mucopolysaccharidosis II), *LIPA* KO (LAL-D), *MAN2B1* KO (α-mannosidosis), *MCOLN1* KO (mucolipidosis IV), *NEU1* KO (sialidosis), *NPC1 KO* (Niemann-Pick disease type C), *PPT1* KO (neuronal ceroid lipofuscinosis 1), *PSAP* KO (combined saposin deficiency), *SMPD1* KO (Niemann-Pick disease type A/B), and *TPP1* KO (neuronal ceroid lipofuscinosis 2). We collected cell pellets and quantified Lyso-Gb3 in the cells by LC-MS/MS. The results showed that Lyso-Gb3 levels were more than three times higher than those in non-edited cells in three cell lines, including *GLA* KO, *GNPTAB* KO, and *PSAP* KO cells. These cells showed a statistically significant or a tendency to increase in Lyso-Gb3 compared to non-edited cells. *NEU1* KO and *NPC1* KO cells showed approximately two-fold increases, and the other cell lines showed no obvious increase in Lyso-Gb3 compared to non-edited cells (Figure 1A).

Lyso-Gb3 has already been established as a useful clinical biomarker for Fabry disease [7,8,9]. Therefore, the detection of increased Lyso-Gb3 in the *GLA* KO cells demonstrates the validity of the model cell and the measuring system.

Prosaposin, encoded by the *PSAP* gene, is the precursor of saposins A, B, C, and D, which are produced by post-translational cleavage [11,12]. Deficiency of PSAP causes combined saposin deficiency, which is a fatal infantile storage disorder with hepatosplenomegaly and severe neurologic symptoms [12,13]. Each saposin is a co-factor for the hydrolytic reaction of lysosomal enzymes. Because SapB supports the degradative functions of α-galactosidase A (encoded by *GLA*), it is conceivable that the loss of prosaposin caused the intracellular accumulation of Lyso-Gb3 [14].

ML II/III is caused by a biallelic mutation in *GNPTAB* [15,16,17], leading to a defect of GlcNAc 1-phosphotransferase that works for the addition of the M6P residues on lysosomal enzymes. M6P residues are necessary for the targeting of lysosomal enzymes to lysosomes [18]. Since α-galactosidase A (encoded by *GLA*) and lysosomal acid lipase (encoded by *LIPA*) are also transported to lysosomes via the M6P-dependent pathway, M6P-modified enzymes are used in enzyme replacement therapy for Fabry and LAL-D. It is speculated that Lyso-Gb3 accumulation in ML II cells is caused by a combined effect of a lack of lysosomal enzymes and the disruption of acidic environments inside lysosomes [2].

The amounts of Lyso-Gb3 were investigated in ML II/III patient specimens. Lyso-Gb3 was significantly elevated in ML II/III patient skin fibroblasts compared with normal skin fibroblasts (Figure 1B). The cellular accumulation of Lyso-Gb3 showed a tendency to decrease (*p* = 0.097) after total lysosomal enzyme supplementation (Figure 1B). These results indicate that the levels of Lyso-Gb3 have biomarker properties that change in response to treatment.

Lyso-Gb3 in patient plasma was measured from residual specimens used for the diagnosis of LSDs. Among the diseases targeted in Figure 1A, LSDs, for which multiple measurement data were obtained, are shown in Figure 1C. Although an increase in Lyso-Gb3 was observed in some Fabry disease patients’ plasma, no clear increase in Lyso-Gb3 was observed in other LSDs measured in this study, such as Gaucher, Krabbe, metachromatic leukodystrophy (MLD), and ML II/III. These data are consistent with a previous report [19]. Due to the limited number of samples analyzed, further analysis is awaited to reach a conclusion. Interestingly, Lyso-Gb3 does not appear to leak into the plasma of ML II/III patients (Figure 1C), although cellular accumulation of Lyso-Gb3 is comparable between the Fabry and ML II/III models. The difference in plasma Lyso-Gb3 in patients with Fabry disease and ML II/III has important implications for the pathomechanism of these LSDs.

Recently, measurements of lysosomal enzyme activity in lymphocytes in dried blood spots have been used in newborn screening of LSDs. B lymphocytes in ML II have been found to have an accumulation of storage material and impaired function [20]. Our results suggest that measurements of Lyso-Gb3 in blood cells may be a biomarker for ML II/III, even though Lyso-Gb3 does not change in plasma.

## 3. Materials and Methods

### 3.1. Knockout (KO) Cell Lines

Lysosome-related genes were knocked out using CRISPR/Cas9 in HeLa Kyoto, a cell line for which whole genome analysis has been performed [21]. HeLa Kyoto cells were originally established at Kyoto University based on canonical HeLa cells, were provided to EMBL, and are now distributed worldwide without any limitation (RRID: CVCL_1922). Plasmids expressing each CRISPR guide RNA and Cas9 protein were prepared based on px458 (pSpCas9(BB)-2A-GFP (PX458)), which was a gift from Feng Zhang (Addgene plasmid #48138; http://n2t.net/addgene:48138 (accessed on 3 October 2017); RRID: Addgene_48138) [22]. Guide sequences for gene targeting were designed using online tools, either the Benchling CRISPR Guide RNA Design Tool (https://www.benchling.com/crispr/ (accessed on 6 July 2020)) or CRISPRdirect (https://crispr.dbcls.jp/ (accessed on 24 June 2022)) (Table 1). The double-stranded DNA coding guide RNA sequences were prepared by annealing synthesized complementary DNA oligo pairs on a thermal cycler after phosphorylation by the T4 polynucleotide kinase (TaKaRa Bio, Shiga, Japan) and then cloned at the *BbsI* site of the px458 plasmid. The targeting plasmid was transfected into HeLa Kyoto cells with the Effectene Transfection reagent (Qiagen, Hilden, Germany). After 48 h of transfection, GFP-positive cells were sorted into 96-well plates as a single cell culture by FACSAria III™ Cell Sorter (BD Biosciences, CA, USA) and expanded. Genomic DNA from each clone was prepared using the QuickExtract™ DNA Extraction Solution (Lucigen, Oxford, UK). The genomic sequence of each targeted site was PCR-amplified with gene-specific primer sets, and genome editing was analyzed by Sanger sequencing. KO clones were characterized by phenotyping either lysosomal enzyme activities, substrate accumulation, or autophagic flux and confirmed by genotyping. The established LSD model cell lines were maintained in Dulbecco’s Modified Eagle Medium (DMEM) (Merck, MA, USA) supplemented with 10% fetal bovine serum FBS, 100 units/mL penicillin, and 100 µg/mL streptomycin (Nacalai-Tesque, Kyoto, Japan). The cell pellets for LC-MS/MS analysis were prepared by harvesting cells from multiple dishes in subconfluent conditions with trypsin–EDTA digestion, followed by several PBS(-) washes.

### 3.2. Patient Specimens

ML II/III patient skin fibroblasts (SFs) and LSD patient plasma were obtained from residual specimens of diagnostic research after obtaining informed consent. The use of the patients’ specimens was approved by the institutional ethics review board (Kawasaki Medical School: approval Number: 5889). Patient SF lines were established from a 5 mm square of buttock skin piece from each patient. The tissue was excised by punching and then shredded with a clean scalpel and scissors. The disrupted skin tissue was directly transferred and cultured in the AmnioMAX-II™ medium (Thermo Fisher, Waltham, MA, USA). After weeks of expansion, grown colonies were dispersed by trypsin–EDTA digestion and thereafter cultured in the normal DMEM as described above in the HeLa Kyoto part. Genotypes of ML II/III patient SFs were #4 c.310C>T (p.Q104X)/c.2522delA (p.K848fs), #5, and #6 c.1120T>C (p.F374L)/c.3565C>T (p.R1189X). For healthy controls, three commercially available normal SFs were purchased from Kurabo (Kurashiki, Japan), Thermo Fisher Scientific Inc. (Waltham, MA, USA), and Lonza (Basel, Switzerland).

In vitro therapeutic intervention for ML II/III SFs via total lysosomal enzyme supplementation was performed as described before [2]. In short, the normal SF was incubated with ammonium chloride, and M6P-tagged lysosomal enzymes exhaled in the culture supernatant were collected. Ammonium chloride was removed using a molecular weight cut-off filter, and the lysosomal enzyme mixture was purified. ML II/III patient SFs were treated with a conditioned medium containing the M6P-tagged lysosomal enzyme mixture for 72 h to replenish lysosomal enzymes.

### 3.3. LC-MS/MS Analysis for Lyso-Gb3

The internal standard reagent was purchased from Avanti Polar Lipid (Alabaster, CA, USA). Methanol, isopropanol, and chloroform of ultra-performance liquid chromatography (UPLC)/MS quality were obtained from FUJIFILM Wako Pure Chemical Corporation (Osaka, Japan). Ultrapure water was obtained from a Milli-Q water system (Millipore, Billerica, MA, USA). Briefly, cell lines were mixed with 1.0 mL of methanol containing the internal standards. The samples were briefly sonicated and incubated on ice for 0.5 h. The samples were then centrifuged for 3 min at 10,000× *g*. Liquid phases were collected in measuring vials. LC-MS/MS analysis was performed using the Xevo TQ-XS mass spectrometer with an ACQUITY UPLC H-Class system (Waters). The lipids were separated on a Waters X-Bridge C18 column (3.5 mm, 150 mm × 1.0 mm internal diameter) at 40 °C using a gradient solvent system as follows: mobile phase A was isopropanol/methanol/water (5/1/4 *v*/*v*/*v*) supplemented with 5 mM ammonium formate and 0.05% ammonium hydroxide (28% in water); mobile phase B was isopropanol supplemented with 5 mM ammonium formate and 0.05% ammonium hydroxide (28% in water) with a flow rate of 80 mL/min. Lyso-Gb3 metabolites were measured using multiple reaction monitoring (MRM) in the positive ion mode. The peak areas of the individual species were normalized to those of internal/surrogate standards, which were added to the samples before lipid extraction. Raw LC-MS/MS data were processed using analytical software (MassLynx 4.2; Waters). The quantification and annotation methods used in this study correspond to “absolute quantification Level 2” and the “Fatty Acyl/Alkyl Level“ defined by the Lipidomics Standard initiative, respectively [23].

## Figures and Tables

**Table 1:** List of CRISPR guide RNA sequences and variants for knockout HeLa cell lines used in this study.

Gene	CRISPR Guide (5′ to 3′)	Clone #1	Clone #2	Clone #3
ARSA	GGGAGTCCCCAAATGGCCCG	delC/del7bp	del13bp/insG	del16bp homo
ARSB	GCTGCGTGTAGTAGTTGTCC	del2/del11bp	delC/delCACC	insC/del8bp
ASAH1	TCAAGATTTATGGTGTACCA	insT homo	del7bp homo	insT/delG
CTSA	CTTTGAGGTAGCCGGAGTAC	del11 homo	del11bp/del23bp	delTC/delCTCC
CTSD	GTCCATCCGCCGGACCATGT	del10bp homo	delCA/del40bp	delT homo
FUCA1	GAACTTGGCTTCGTCGAACC	delT/insG	delTC/del8bp	insT homo
GAA	AGGGATGTAGCAACAGCCGC	delGC homo	CTGT > GCTGC homo	insG/delCTG
GALC	GTAATTTACTAGAAGTCGGG	insT/delC	insG homo	insGA homo
GBA	AGACCAATGGAGCGGTGAAT TGTGGTGAGTACTGTTGGCG *	del4bp/del5bp	insA/insCC	insC/delA
GLA	GCTAGCTGGCGAATCCCATG	delG homo	delG homo	delG/insGG
GLB1	CCCGTGTGCCCCGCTTCTAC	del8/del19bp	insAT/del19bp	delACT/del26bp
GNPTAB	ACACGTAGAGCCCATACCTG	delGT/del8bp	delG homo	delAG/del12bp
GUSB	GTGGTACCGGCGGCCGCTGT	delGC homo	insT/insG	insC homo
HEXA	GTTGTCTCTGTAGTCACACC	delCC/del13bp	insC/delTCAC	delAC/del5bp
HEXB	GAGGGGCCCGCCGTGGAATT	delT/del8bp	delT/insT	delT homo
IDS	GAACGTTCTTCTCATCATCG	insA homo	delT/delC	delA/delTC
LIPA	TCCCATGAGGAATTCGGTTA	delC homo	del13bp homo	insC/del5bp
MAN2B1	ACGTAAATGAAGCGACGGGT	delCC homo	insCG/del5	delC homo
MCOLN1	AGTATTTGAGACGACGGCGA	delC homo	delC homo	delC/delGC
NEU1	CCAAGTTCATCGCCCTGCGG	del5 homo	delG/delG	delC homo
NPC1	GCGCTGGACACAGTAGCAGC	del12bp homo	insT/del8bp	del7bp homo
PPT1	GCAGCAAGGCTACAATGCTA	delTG/del5bp	delTG homo	del5bp/del8bp
PSAP	TGAAGACGGCGTCCGACTGC	insT/delAC	del7bp homo	delT/ del5bp
SMPD1	GTTCTTTGGCCACACTCATG	insC/del14bp	insC homo	insT/del10bp
TPP1	TGTGGAAAGACTCTCGGAGC	delG/delGG	delGG homo	delGG/delTCGG

* Two different guides were used.

## Data Availability

The datasets generated and/or analyzed during the current study are not publicly available but are available from the corresponding author upon reasonable request.

## Abbreviations

The following abbreviations are used in this manuscript:

LSD	lysosomal storage disease
Lyso-Gb3	globotriaosylsphingosine
ML II/III	mucolipidosis II/III
LAL-D	lysosomal acid lipase deficiency
M6P	mannose 6-phosphate
Gb3	globotriaosylceramide
KO	knockout
SF	skin fibroblast
ERT	enzyme replacement therapy
